# Angiopoietin-1 Upregulates Cancer Cell Motility in Colorectal Cancer Liver Metastases through Actin-Related Protein 2/3

**DOI:** 10.3390/cancers14102540

**Published:** 2022-05-21

**Authors:** Miran Rada, Audrey Kapelanski-Lamoureux, Migmar Tsamchoe, Stephanie Petrillo, Anthoula Lazaris, Peter Metrakos

**Affiliations:** Cancer Research Program, Research Institute of the McGill University Health Centre, Montreal, QC H4A 3J1, Canada; audrey.kapelanski-lamoureux@mail.mcgill.ca (A.K.-L.); migmar.tsamchoe@mail.mcgill.ca (M.T.); stephanie.petrillo@muhc.mcgill.ca (S.P.); anthoula.lazaris@mail.mcgill.ca (A.L.)

**Keywords:** CRCLM, angiogenesis, vessel co-option, Ang1, ARP2/3, Tie2, PI3K/AKT

## Abstract

**Simple Summary:**

Vessel co-option has been recognized as a mechanism associated with resistance to anti-angiogenic treatment in colorectal cancer liver metastases (CRCLMs). Recently, we reported that Angiopoietin-1 (Ang1) stimulates vessel co-option in CRCLM through an unknown mechanism. In this manuscript, we found the molecular pathways that mediate the function of Ang1 in CRCLM. We showed that Ang1 induces the expression levels of actin-related protein 2/3 (ARP2/3) in the cancer cells via various mechanisms. Importantly, different studies have shown that high levels of ARP2/3 in cancer cells are essential for the formation of vessel co-opting CRCLM tumours. Highlighting these pathways is an important step to identify therapeutic strategies to overcome vessel co-option and resistance to anti-angiogenic therapy in CRCLM.

**Abstract:**

Resistance to anti-angiogenic therapy is a major challenge in the treatment of colorectal cancer liver metastases (CRCLMs). Vessel co-option has been identified as a key contributor to anti-angiogenic therapy resistance in CRCLMs. Recently, we identified a positive correlation between the expression of Angiopoietin1 (Ang1) in the liver and the development of vessel co-opting CRCLM lesions in vivo. However, the mechanisms underlying its stimulation of vessel co-option are unclear. Herein, we demonstrated Ang1 as a positive regulator of actin-related protein 2/3 (ARP2/3) expression in cancer cells, in vitro and in vivo, which is known to be essential for the formation of vessel co-option in CRCLM. Significantly, Ang1-dependent ARP2/3 expression was impaired in the cancer cells upon Tie2 or PI3K/AKT inhibition in vitro. Taken together, our results suggest novel mechanisms by which Ang1 confers the development of vessel co-option in CRCLM, which, targeting this pathway, may serve as promising therapeutic targets to overcome the development of vessel co-option in CRCLM.

## 1. Introduction

Colorectal cancer (CRC) is one of the leading causes of tumour-related morbidity and mortality worldwide [1]. The majority of CRC patients will form liver metastases (LM), which account for the most common cause of mortality in CRC patients [2]. Surgical resection can extend median survival and even offers a potential cure in eligible colorectal cancer liver metastasis (CRCLM) patients [3,4]. However, 80% of CRCLM patients have unresectable tumours [5]. Therefore, the efforts are directed towards downsizing with chemotherapy and targeted therapies, including anti-angiogenic agents (e.g., Bevacizumab) to convert the tumours into a resectable state [5,6,7].

CRCLM tumours are mainly divided into two distinct histopathological growth patterns (HGPs): desmoplastic HGP (DHGP) and replacement HGP (RHGP) [8,9]. The desmoplastic lesions have a desmoplastic rim that separates the cancer cells from surrounding the liver tissue; the cancer cells and hepatocytes do not make contact and they acquire their blood supply via sprouting angiogenesis [9,10,11]. In contrast, the cancer cells in replacement lesions overlap with the hepatocytes at the tumour–liver interface and invade the liver parenchyma to replace the hepatocytes and co-opt the pre-existing liver sinusoidal vessels to obtain their blood supply instead of stimulating angiogenesis [9,12,13,14]. Since anti-angiogenic agents were designed to target only new blood vessel growth, vessel co-option has been implicated as a mechanism of intrinsic and/or acquired resistance to anti-angiogenic therapy in different types of cancer, including CRCLM [11,15,16]. In this context, it has been reported that the therapeutic efficacy of anti-angiogenic therapy (e.g., bevacizumab) in CRCLM patients with vessel co-opting tumours is modest in comparison to patients with angiogenic lesions [9,12,13,17]. Therefore, deciphering the molecular mechanism(s) vessel co-option and developing therapeutics that would target the mechanism(s) has become of the utmost importance.

Angiopoietin-1 (Ang1) is a secreted protein ligand of the tyrosine kinase receptor (Tie2), and it is involved in vascular and hematopoietic development [18]. Ang1 overexpression has been linked to tumour dissemination, metastasis [19] and tumour angiogenesis [20] in various cancers. However, its role in vessel co-option is poorly understood. Recently, we found that Ang1 is a key mediator of vessel co-option in CRCLM [17]. Accordingly, Ang1 deficiency in the host liver abrogates tumour progression and development of vessels co-opting liver metastatic lesions through unknown mechanisms [17].

The actin-related protein 2/3 (ARP2/3) complex is composed of seven subunits: ACTR2, ARP3, ARPC1, ARPC2, ARPC3, ARPC4 and ARPC5 [21,22]. ARP2/3 has been reported as a mediator of tumour invasion and progression in a variety of cancers [10]. Additionally, ARP2/3 has been shown to contribute to the generation of vessel co-option in CRCLM [9]. Accordingly, silencing ARPC3, a subunit of the ARP2/3 complex, in colorectal cancer (HT29) cells decreased the generation of vessel co-opting CRCLM tumours in vivo [9].

In the current manuscript, we suggest the molecular mechanisms by which Ang1 stimulates the formation of vessel co-opted CRCLM lesions. Our data suggested a positive correlation between Ang1 and ARP2/3 expression in the cancer cells, in vitro and in vivo, which is mediated by the Tie2/PI3K-AKT pathway.

## 2. Materials and Methods

### 2.1. Patient Samples

McGill University Health Centre Institutional Review Board approved the protocol, and all patients (*n* = 3) provided written informed consent.

### 2.2. Cell Cultures

Human colon cancer cell lines (COLO320DM and SW620) derived from the human large intestine, MC38 colon cancer cells derived from C57BL/6 mouse and HEK293T (Human Embryonic Kidney) cells were used for the current study. The cell lines were a gift, kindly provided by Drs Alex Gregorieff and Pnina Brodt (Cancer Research Program, McGill University). The cells were seeded in DMEM media (Wisent Inc., St-Bruno, QC, Canada, #319-005-CL) supplemented with 10% FBS (Wisent Inc., St-Bruno, QC, Canada, #085-150) and 100 IU/mL streptomycin/ penicillin (Wisent Inc., St-Bruno, QC, Canada, 450-201-EL), and incubated at 37 °C with 5% CO_2_.

Prior to treatment with recombinant Ang1 (Peprotech, Cranbury, NJ, USA, #130-06), the medium was aspirated and replaced by serum-free DMEM media. The treated cells were incubated for 24 h at 37 °C with 5% CO_2_. We also treated the cells with either 0.4 μM BAY-826 (Tocris Bioscience, Toronto, ON, Canada, #6579) or 2 μM LY294002 (Selleckchem, Burlington, ON, Canada, #S1105) for 24 h to block Tie2 or PI3K/AKT pathway in the cells. We also treated the control cells with DMSO (Tocris Bioscience, Toronto, ON, Canada, # 3176) based on the concentration of the used inhibitors. Therefore, we treated the control cells with 0.004% of DMSO for the Tie2 blocking experiments, while we used 0.02% of DMSO for treating the control cells in the PI3K/AKT inhibition experiments.

### 2.3. Lentiviral ShRNA Knockdown

In this study, we used the following lentiviral constructs, purchased from Sigma Aldrich (Oakville, ON, Canada): Scrambled: SHC016 (GCGCGATAGCGCTAATAATTT), ARPC3 #1: TRCN0000333048 (CTGATACCAAACTCATCGGAA), ARPC3#2: TRCN0000333049 (TGAAGCTGATAGGACCTTGAT), Tie2 #1: TRCN0000023554 (CGCATCAAGAAGGATGGGTTA) and Tie2 #2: TRCN0000023556 (GCCTTTCAACATTTCCGTCAA).

We generated the lentiviruses in HEK293T cells using the Calcium-Phosphate-based method. To transduce the cancer cells, we incubated the cells using supplemented media with lentiviruses and 8 µg/mL of polybrene (Sigma Aldrich, Oakville, ON, Canada, #107689) at 37 °C with 5% CO_2_ for 72 h. The transduced cells were selected by incubating the cells in a regular DMEM growth medium containing 1 µg/mL of Puromycin (Wisent Inc., St-Bruno, QC, Canada, #450-162-XL) for 15 days.

### 2.4. Immunoblotting

The cells were harvested, and protein lysates were prepared for immunoblotting using RIPA buffer (Sigma Aldrich, Oakville, ON, Canada, #R0278). Briefly, the media were aspirated, and the cells were washed by PBS, trypsinized, harvested and resuspended in RIPA buffer. The cells were sheared mechanically by passing through a 25-gauge needle 15 times. The protein content of the samples was determined by BCA Protein Assay Kit (Thermo Scientific, Saint-Laurent, QC, Canada, #23225). The generated lysates were combined with sample 4× Laemmle sample buffer (Biorad, Saint-Laurent, QC, Canada, #1610747), boiled for 4 min and then subjected to 10 % SDS-polyacrylamide electrophoresis. Finally, the blots were visualized with ECL detection system (Thermo Scientific, Saint-Laurent, QC, Canada, #32106) and the blots were visualized using ECL and LAS4000 imaging systems. The quantification of the band’s intensity was performed with Image J software (Table S1). The following primary antibodies were used: GAPDH 1:2000 (Abcam, Waltham, MA, USA, #ab9485), ARP2/3 1:1000 (Millipore, Etobicoke, ON, Canada, #MABT95), Tie2 1:1000 (Abcam, Waltham, MA, USA, #ab221961) and p-Akt (S473) 1:500 (Cell signaling, Whitby, ON, Canada, #9271S). All uncropped Western blots can be found in Supplementary Figures S1–S4.

### 2.5. Immunohistochemical Staining

We performed IHC staining for formalin-fixed paraffin-embedded (FFPE) specimens. We cut sections (4 mm thick), mounted on slides and baked them overnight at 37 °C. We baked the section at 60 °C for 1 h and performed staining using the following antibodies: ARP2/3 1:300 (Bioss, Laval, QC, Canada, #bs-12524R) and Ang1 1:500 (Abcam, Waltham, MA, USA, # ab102015). Briefly, the sections were deparaffinized by xylene (Leica, Concord, ON, Canada #3803665) and hydrated with ethanol (Leica, Concord, ON, Canada #3803686) and distilled water. Then, we performed antigen retrieval using Sodium Citrate (10 mM, Ph = 6.0) and blocked endogenous peroxidase activity using hydrogen peroxide (Dako, Burlington, ON, Canada, #S2003). Prior to incubation with the antibodies, we blocked the sections by incubating with 5% goat serum for 1 h at room temperature. Then, we removed the blocking buffer and added the designated primary antibody that was diluted in 5% goat serum and incubated overnight at 4 °C. Next day, the sections were washed thrice with 1× PBS supplemented with Tween (Thermo Fisher, Waltham, MA, USA, #BP-337) and incubated with a secondary antibody (Dako, Anti-Mouse: #K4001; Anti-Rabbit: #K4003) for 1 h at room temperature. After washing, the sections were incubated with diaminobenzidine (DAB) substrate (Dako, Burlington, ON, Canada, #K3468) for 5 min. The sections were washed, stained with Harris Modified Hematoxylin (Thermo Fisher, Saint-Laurent, QC, Canada, #SH26-4D) for 30 s, washed with distilled water, dehydrated using ethanol and xylene, and covered with a coverslip. The slides were then scanned and analyzed with Aperio ImageScope ver.11.2.0.780 software as described in previous publications [8,12,23]. The positivity values are presented in Table S2.

### 2.6. Immunofluorescence Staining

We performed co-immunostaining for FFPE sections as described in previous publications [10,24]. Briefly, we performed deparaffinization, hydration, antigen retrieval and blocking of endogenous peroxidase activity and non-specific binding as described in the immunohistochemical staining section above. We prepared the primary antibodies in 5% goat serum as follows: ARP2/3 1:300 (Bioss, Laval, QC, Canada, #bs-12524R) and Tie2 1:400 (Invitrogen, Burlington, ON, Canada, # PA5-28582). The sections were incubated with the primary antibodies at 4°C overnight. Next day, the sections were washed and incubated with a mixture of secondary antibodies for 2 h. The secondary antibody mixture was composed of 5% goat serum containing the following antibodies: Alexa Flour 594 goat anti-rabbit IgG 1:500 (Invitrogen, Burlington, ON, Canada, #A11037) and Alexa Flour 488 goat anti-mouse IgG 1:500 (Invitrogen, Burlington, ON, Canada, #A10680). Next, the sections were washed and incubated with DAPI (1:1000 in 1× PBS) for 10 min. The sections were then protected with coverslips using ProLong Mountant (Thermo Fisher Scientific, Saint-Laurent, QC, Canada, #P36934).

### 2.7. Scratch Assay

We performed the scratch assay as described in Rada et al. [24] and Ibrahim et al. [17]. Briefly, the cells (0.5 × 10^6^) were cultured in 6-well plates and incubated at 37 °C with 5% CO_2_. Once the cells grew to 80% confluence, the scratch was introduced using a p200 pipette tip. The cells were then washed with 1× PBS (Wisent Inc., St-Bruno, QC, Canada, #311-010-CL) and the stripped areas were imaged (time: 0 h). We removed the media, washed with 1× PBS and added a serum-free DMEM medium (Wisent Inc., St-Bruno, QC, Canada, #319-005-CL) containing recombinant Ang1 (Peprotech, Cranbury, NJ, USA, #130-06) and incubated at 37 °C with 5% CO_2_. 24 h later, the medium was removed and the cells were washed with PBS followed by imaging (time 24 h). This experiment was performed in triplicate (*n* = 3). To analyze the wound opening, we used ImageJ software (NIH, Bethesda, MD, USA).

### 2.8. Statistical Analysis

We used GraphPad Prism software version 8.0 (GraphPad Software, La Jolla, CA, USA) for statistical analyses. The data are presented as standard deviation. Statistical significance between the two groups was analyzed using unpaired Student’s *t*-test. The correlation between two proteins and R-value was assessed using Pearson correlation. *p*-values of <0.05 were considered to be significant.

## 3. Results

### 3.1. Ang1 Induces Cancer Cell Motility through ARP2/3

Our group previously identified that co-culturing mouse colorectal cancer (MC38) cells with mouse primary hepatocytes results in increasing cancer cell migration in vitro [17]. Strikingly, the effect of hepatocytes on cancer cell migration was impaired when we co-cultured cancer cells with isolated primary hepatocytes from Ang1 knockout (Ang1-KO) mice [17], suggesting that Ang1 contributes to cancer cell motility [17]. However, the mechanism behind the regulatory role of Ang1 in cancer cell motility is unknown.

**Figure 1:**
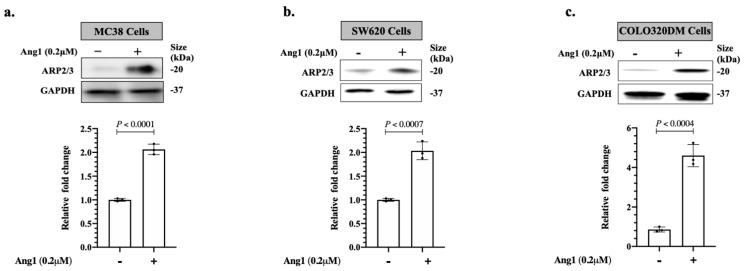
Ang1 induces ARP2/3 expression in colorectal cancer cells in vitro. (**a**–**c**) Western blotting of ARP2/3 expression in colorectal cancer (COLO320dm, SW620 and MC38) cells in the presence or absence of Ang1 (top panel). The intensity of ARP2/3 bands (*n* = 3) were quantified and normalized against GAPDH using ImageJ and represented as a fold change (bottom panel).

**Figure 2:**
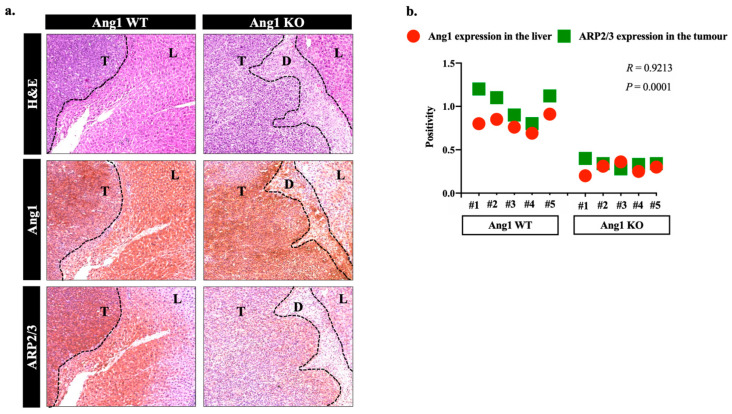
Ang1 presence is essential for ARP2/3 expression in the cancer cells in vivo. (**a**) RepreScheme 1 and ARP2/3 staining of tumour sections generated by intrasplenic injection of mouse colorectal (MC38) cancer cells into wild type (Ang1 WT) and Ang1 knockout (Ang1 KO) mice (left panel). D: Desmoplastic ring, L: Liver tissue, T: Tumour. (**b**) Represents the Correlation between Ang1 expression in the liver tissue and ARP2/3 expression in the tumour cells using Pearson correlation analysis.

Since ARP2/3 plays a key role in cancer cell motility [10], we decided to investigate the influence of Ang1 on ARP2/3 expression in various colorectal cancer cells, including MC38, SW620 and COLO320DM in vitro using recombinant Ang1 [17]. The results suggested Ang1 as a positive regulator of ARP2/3 expression in the cancer cells (Figure 1). To further validate our results and evaluate the effect of Ang1 on the expression of ARP2/3 in the cancer cells in vivo, we performed immunohistochemical staining on the specimens that were generated from the publication of Ibrahim et al. [17] using Ang1 and ARP2/3 antibodies (Figure 2a). Indeed, the expression levels of ARP2/3 in the metastatic cancer cells were significantly correlated with the presence of Ang1 in the liver tissue (Figure 2b).

**Figure 3:**
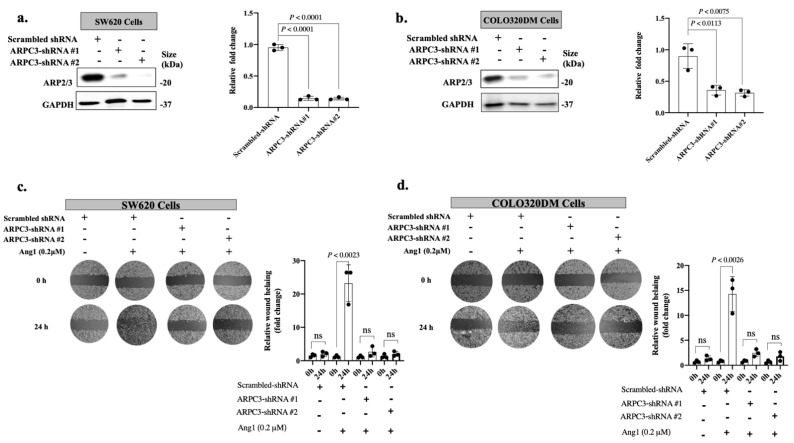
ARP2/3 mediates Ang1-driven cancer cell motility. (**a**,**b**) Western blotting of ARP2/3 expression in SW620 or COLO320dm cancer cells expressing shRNA-scrambled or shRNA-ARPC3. The right panels show the intensity of ARP2/3 bands that were quantified and normalized against GAPDH using ImageJ and represented as a fold change (*n* = 3). (**c**,**d**) Representative scratch assay in SW620 or COLO320dm cells expressing shRNA-scrambled or shRNA-ARPC3 upon exposure to Ang1. The right panels show the corresponding wound healing ratio shown in fold change (*n* = 3). Data are presented as the mean ± SD. ns = Not significant.

To determine whether ARP2/3 mediates Ang1-driven cancer motility, we knocked down ARPC3, a subunit of ARP2/3, using ARPC3-specific shRNA in SW620 and COLO320DM cancer cells (Figure 3a,b), followed by scratch assay [17,24,25,26] (Figure 3c,d). The data showed significant elevation in the migratory capacity of cancer cells upon Ang1 exposure, whilst the effect of Ang1 on cancer cell motility was abolished in the cancer cells expressing shRNA-ARPC3. Altogether, our data proposed ARP2/3 as a mediator of Ang1-driven motility in cancer cells.

### 3.2. Ang1 Regulates ARP2/3 Expression through Tie2-PI3K/AKT Pathway

**Figure 4:**
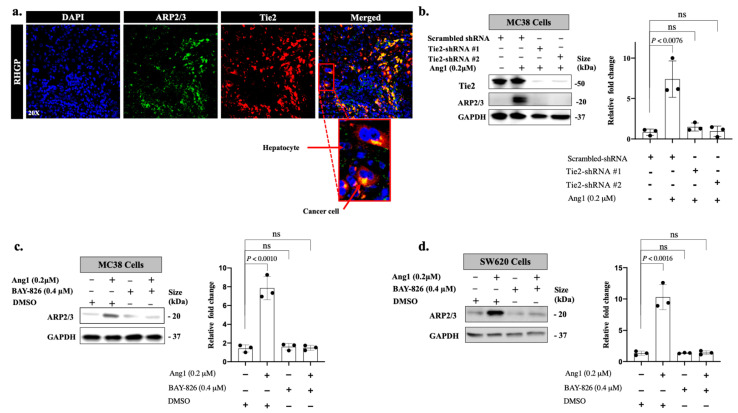
Tie2 mediates Ang1-dependent ARP2/3 expression in the cancer cells. (**a**) Representative images of coimmunostaining for showing ARP2/3 (green) and Tie2 (red) on FFPE tissue sections of CRCLM resected from chemonaïve patients. The bottom panel represents the cancer cells at the invading front that are in direct contact with hepatocytes. (**b**) Immunoblotting of ARP2/3 expression in MC38 colorectal cancer cells expressing shRNA-Scrambled or shRNA-Tie2. The right panel shows the intensity of ARP2/3 bands that were quantified and normalized against GAPDH using ImageJ and represented as a fold change (*n* = 3). (**c**,**d**) Left panels show the effect of Tie2 inhibitor (BAY-826) on ARP2/3 expression in MC38 and SW620 cancer cells upon Ang1 exposure, respectively. Right panels show the intensity of ARP2/3 bands that were quantified and normalized against GAPDH using ImageJ and represented as a fold change (*n* = 3). Data are presented as the mean ± SD. ns = Not significant.

Having identified a link between Ang1 and ARP2/3, we further evaluated the signaling pathway involved in Ang1-dependent ARP2/3 expression. Ang1 is known as a Tie2 agonist, which promotes phosphorylation of Tie2 to activate its downstream pathways [27,28]. Tie2 was initially found to be overexpressed in tumour vessels, while recent studies reported Tie2 expression in cancer cells [17,29]. Since Tie2 expression is upregulated in the cancer cells of vessel co-opting CRCLM lesions [17], we speculated that Tie2 may facilitate Ang1-dependent ARP2/3 expression. Firstly, we examined the colocalization of ARP2/3 and Tie2 in vessel co-option tumours by coimmunostaining using anti-ARP2/3 and anti-Tie2 antibodies. Indeed, our results showed colocalization of both proteins in the cancer cells; specifically, the cancer cells at the leading edge of the tumour lesions that are in close proximity with hepatocytes (Figure 4a). Next, we addressed the possibility of Tie2 involvement in Ang1-dependent ARP2/3 using Tie2-silenced MC38 cancer cells (Figure 4b). Remarkably, the absence of Tie2 significantly reduced the effect of Ang1 on ARP2/3 expression. To further validate our results, we cultured MC38 and SW620 colorectal cancer cells with recombinant Ang1 for 24 h in the presence or absence of pharmacological inhibition of Tie2 (BAY-826) [30], followed by Western blotting to evaluate ARP2/3 expression. As shown in Figure 4c,d, the presence of BAY-826 resulted in significant impairment in Ang1-dependent expression of ARP2/3 in the cancer cells.

**Figure 5:**
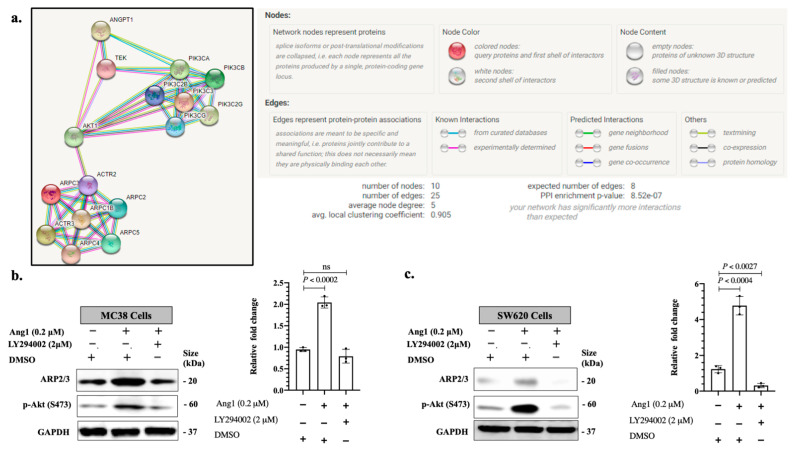
PI3K/AKT pathway contributes to Ang1-dependent ARP2/3 expression. (**a**) Protein interaction networks generated using STRING database version 11 showing the interaction between Ang1 (Angpt1), TIE2 (Tek), PI3Ks (PI3K), Akt (AKT1) and ARP2/3 subunits. (**b**,**c**) Western blotting of ARP2/3 expression in exposed MC38 or SW620 cancer cells to recombinant Ang1 in the presence or absence of PI3K/AKT inhibitor (LY294002). Right panels show the intensity of ARP2/3 bands that were quantified and normalized against GAPDH using ImageJ and represented as a fold change (*n* = 3). Data are presented as the mean ± SD. ns = Not significant.

The Phosphoinositide 3-kinases (PI3K)/Protein kinase B (AKT) signaling pathway has been reported as a key mediator for many receptors, including Tie2 [31,32]. Intriguingly, STRING protein interaction analysis [33] suggested PI3K/AKT pathway involvement in Tie2-ARP2/3 interactions (Figure 5a). Consequently, we decided to explore the role of PI3K/AKT in Tie2-dependent ARP2/3 expression. We exposed MC38 and SW620 cancer cells to recombinant Ang1 upon presence or absence of PI3K/AKT inhibitor (LY294002) [34,35,36]. As shown in Figure 5b,c, the effect on Ang1 in ARP2/3 expression was attenuated upon treatment with 2 µM of LY294002, indicating the involvement of the PI3K/AKT signaling pathway in Ang1-dependent ARP2/3 expression. Collectively, our results imply that Ang1 induces cancer cell motility through ARP2/3 and it is mediated by Tie2-PI3K/AKTsignaling cascade.

## 4. Discussion

During the last several decades, angiogenesis has been regarded as the most important process by which tumours vascularize themselves, while alternative vascularization mechanisms were ignored [37]. Alternative vascularization mechanisms include vessel co-option [9,16], increased pericyte coverage [38], vasculogenic mimicry [39], lysosomal sequestration [40] and glycosylation-dependent angiogenesis [41].

In vessels co-opting CRCLM tumours, the cancer cells meet their metabolic demands without the generation of new vessels [9,12]. The cancer cells migrate and infiltrate the surrounding liver tissue space between pre-existing vessels, ultimately leading to the incorporation of pre-existing vessels into the tumour [8,14,17]. Vessel co-option has emerged as the main alternative vascularization pathway that mediates the failure of anti-angiogenic treatment (e.g., Bevacizumab) in CRCLM [9,42,43]. Moreover, we and others have demonstrated that vessel co-opting CRCLMs are less responsive to chemotherapy [9,16]. Therefore, the CRCLM patients with predominantly angiogenic metastasis receiving neoadjuvant Bevacizumab plus chemotherapy have more than double the 5-year overall survival compared to patients with co-opting tumours who received the same treatment [9,11].

The role of angiopoietin proteins in vessel co-opting tumours has been poorly investigated. Recently, we noticed Ang1 overexpression in vessel co-opting tumours, and specifically, the hepatocytes of the normal adjacent liver that are in very close proximity with cancer cells [17]. Importantly, splenic injection of MC38 into wild-type C57B/6 mice produced only co-opting liver metastatic lesions, while only angiogenic tumours were observed in the Ang1 knockout C57B/6 mice [17]. These results provide evidence that high levels of Ang1 in the host liver are essential to support liver metastatic tumours with vessel co-option and its inhibition favours the formation of angiogenic-driven liver metastases. However, the molecular mechanisms underlying Ang1’s role in the development of co-opted liver metastasis are, as yet, unclear.

Ang1 is thought to support tumour growth mainly through its function in the early stages of vascular activation [44]. However, our results suggest that Ang1 is a positive regulator of cancer cell motility in vitro and in vivo. There are very few publications available that investigated the role of Ang1 in cancer cell motility. The majority of these studies mainly focused on the function of intracellular Ang1 and disregarded the effect of stromal Ang1. In this context, Yang et al. [45] reported Ang1 as an inducer of cancer (HeLa, SiHa, and C33a) cell migration and invasion in vitro, and its downregulation by siRNA significantly attenuated their migration and invasion compared with respective controls. Similar results were obtained in papillary thyroid carcinoma cell lines [32].

Ang1 mainly interacts with cells through Tie2, where its binding activates autophosphorylation of the Tie2 kinase domain and triggers various biological processes [19,27]. Tie2 is originally described as a protein that is predominantly expressed in the endothelial cells and its role in endothelial cell sprouting and vessel growth has been intensively studied in a variety of tumours [17,29]. However, recent studies found that Tie2 expression and activity are not limited to the vasculatures alone, but also expressed by cancer cells and other cell types within the tumour microenvironment, including tumour-associated macrophages in glioblastomas, ovarian, thyroid, gastric and breast cancers [29,46]. We previously reported a significant upregulation of Tie2 in the cancer cells of vessel co-opting CRCLM lesions compared to their angiogenic counterparts [17], while we did not know what their role in vessel co-option is. Our in-vitro data suggested Tie2 as a key mediator in the Ang1-ARP2/3 pathway in colorectal cancer cells. Indeed, overexpression of ARP2/3 is associated with metastases and poor prognosis in various cancers, including CRCLM [9,10,22,24]. In support of our data, high Tie2 expression in cancer cells has been shown to be associated with increased metastases and shorter overall survival in ovarian cancer [47]. Also, upregulation of Tie2 in gastric cancer cells has been linked to higher recurrence rates and poor prognosis [48]. Furthermore, Hossain et al. [49] showed a positive correlation between Tie2 expression and the degree of increasing malignancy in human gliomas. Significantly, their results also revealed that Tie2 overexpression confers a radioresistant phenotype in gliomas via a nonhomologous end-joining mechanism of DNA repair [49]. Since there is some evidence that CRCLM lesions have a relatively radioresistant phenotype [50], it would be interesting to explore the role of Tie2 in radioresistant CRCLM lesions.

Various studies reported that induction of the PI3K/AKT pathway accelerates cancer cell motility and metastases through different mechanisms [51,52]. Our data also suggest PI3K/AKT as a mediator of Ang1-Tie2-driven ARP2/3 expression. Of note, Ang1/Tie2 has also been identified as an upstream activator of PI3K/AKT in endothelial cells [53] and vascular smooth muscle cells [54].

## 5. Conclusions

**Figure 6:**
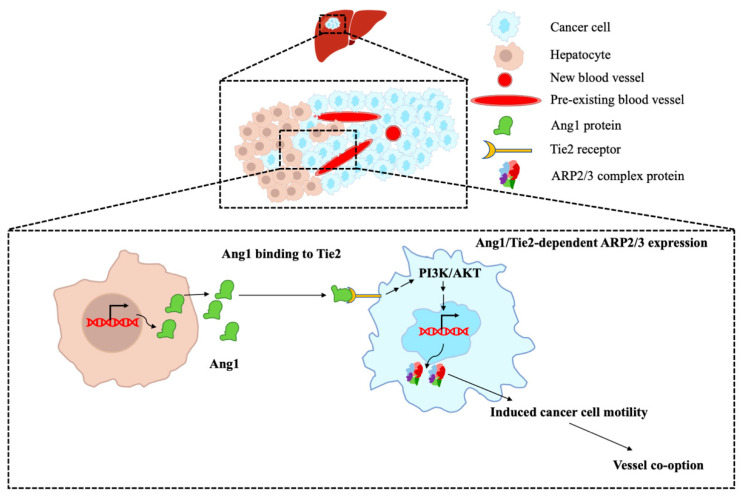
The molecular mechanism of Ang1 function in vessel co-opting CRCLM lesions. Schematic representation of key findings in the current study. The hepatocytes of vessel co-opting lesions express high levels of Ang1. The secreted Ang1 by hepatocytes interacts with the cancer cells through Tie2, which activates PI3K/AKT followed by ARP2/3 expression, respectively. Upregulation in ARP2/3 expression increases cancer cell motility and allows them to infiltrate the liver tissue to obtain blood supply by hijacking the pre-existing vessels (vessel co-option).

In conclusion, our data suggest a mechanistic pathway by which Ang1 confers vessel co-option in CRCLM (Figure 6). Accordingly, Ang1 acts as a positive regulator of ARP2/3 expression in CRCLM cancer cells, which is known to be essential for the formation of vessel co-option [9,10]. However, further studies are required to identify other molecules that are involved in this mechanism. 

## Data Availability

The data presented in this study are available within the article.

## Supplementary Materials

The following supporting information can be downloaded at: https://www.mdpi.com/article/10.3390/cancers14102540/s1. Figure S1: Original uncropped Western blots of Figure 1; Figure S2: Original uncropped Western blots of Figure 3; Figure S3: Original uncropped Western blots of Figure 4; Figure S4: Original uncropped Western blots of Figure 5; Table S1: The intensity ratio of Western blot bands; Table S2: The positivity of the IHC images.
