# Supplementary material for: Angiopoietin-1 Upregulates Cancer Cell Motility in Colorectal Cancer Liver Metastases through Actin-Related Protein 2/3
Source: Cancers (Basel). 2022 May 21;14(10):2540. doi: 10.3390/cancers14102540 (PMC9139616; doi:10.3390/cancers14102540)
Supplement: Supplementary file 1 [file cancers-14-02540-s001.zip › cancers-1719066-supplementary.pdf]

Supplementary Materials

Angiopoietin-1 Upregulates Cancer Cell Motility in Colorectal Cancer Liver Metastases through Actin-Related Protein 2/3

Miran Rada, Audrey Kapelanski-Lamoureux, Migmar Tsamchoe, Stephanie Petrillo, Anthoula Lazaris and Peter Metrakos

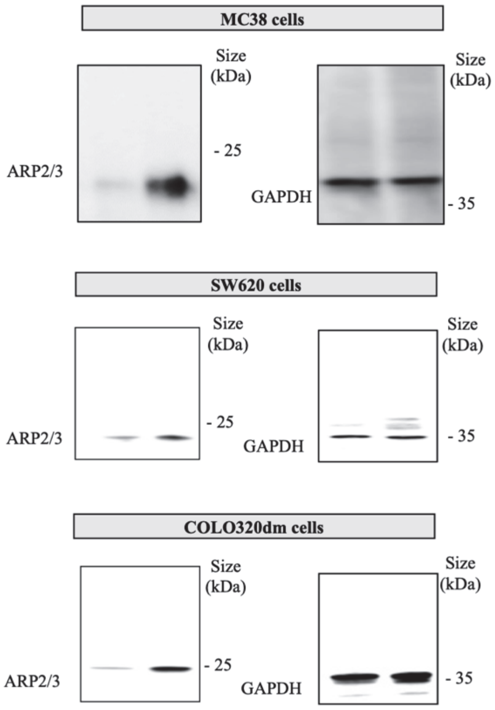

Figure S1. Original uncropped Western blots of Figure 1.

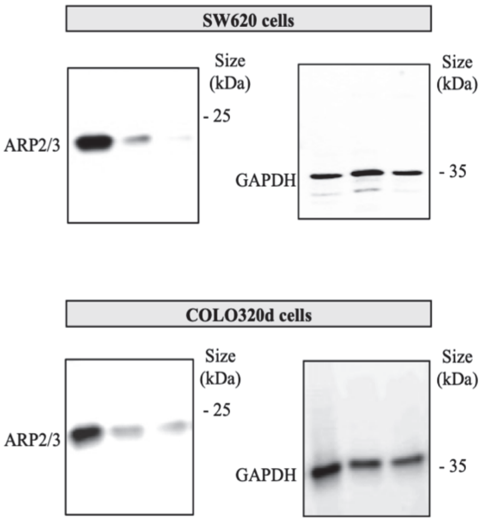

Figure S2. Original uncropped Western blots of Figure 3.

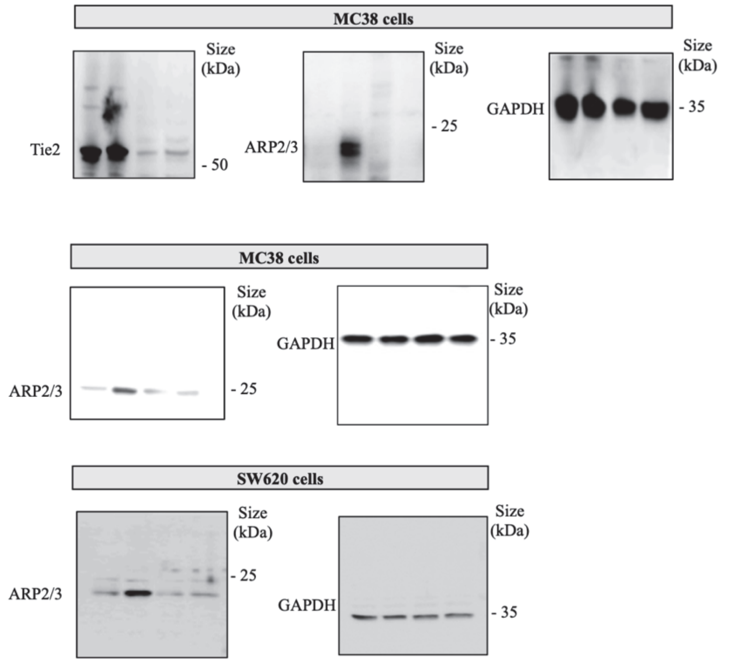

Figure S3. Original uncropped Western blots of Figure 4.

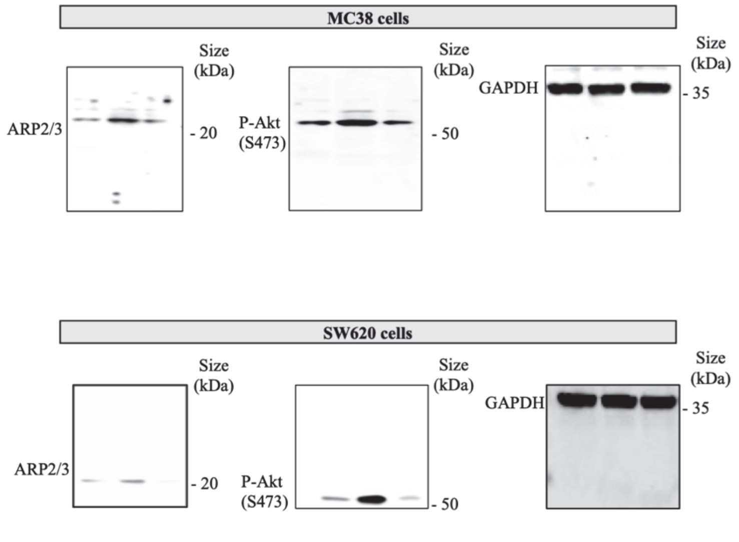

Figure S4. Original uncropped Western blots of Figure 5.

Table S1. The intensity of Western blot bands.

Figure 1a	1	1.956		
	0.968	2.052		
	1.034	2.179		
Figure 1b	1	2.245		
	1.09	1.881		
	0.921	1.977		
Figure 1c	1	5.24		
	0.819	4.18		
	0.746	4.382		
Figure 3a	1	0.126	0.165	
	0.95	0.182	0.128	
	0.91	0.124	0.127	
Figure 3b	1	0.394	0.33	
	1.031	0.413	0.347	
	0.673	0.267	0.256	
Figure 4b	1	10	2.05	1.18
	1.12	6.16	1.27	0.25
	0.4	6.04	1.13	1.46
Figure 4c	1	9.274	1.775	1.526
	1.53	7.34	1.22	1.218
	1.748	6.956	1.839	1.741
Figure 4d	1	12.593	1.437	1.41
	1.672	9.382	1.293	1.732
	1.349	8.902	1.384	1.119
Figure 5b	1	1.975	0.972	
	0.936	1.962	0.666	
	0.907	2.192	0.739	
Figure 5c	1	5.318	0.284	
	1.392	4.714	0.205	
	1.302	4.293	0.45	

Table S2. The expression levels of Ang1 and ARP2/3.

	**Ang1 expression**	
	**Ang1 WT**	**Ang1 KO**
Mouse #1	0.8	0.2
Mouse #2	0.85	0.31
Mouse #3	0.76	0.36
Mouse #4	0.69	0.25
Mouse #5	0.91	0.3
	**ARP2/3 expression**	
	**Ang1 WT**	**Ang1 KO**
Mouse #1	1.2	0.4
Mouse #2	1.1	0.34
Mouse #3	0.9	0.28
Mouse #4	0.8	0.33
Mouse #5	1.12	0.34
